# Thermal conversion of irradiated LLDPE waste into sustainable sponge-like compounds: a novel approach for efficient trace-level oil–water removal

**DOI:** 10.1038/s41598-024-55401-1

**Published:** 2024-02-28

**Authors:** Mohamed Mohamady Ghobashy, H. M. Gayed

**Affiliations:** Radiation Research of Polymer Chemistry Department, National Centre for Radiation Research and Technology, Egyptian Atomic Energy Authority (EAEA), Cairo, Egypt

**Keywords:** Pyrolysis, Plastic, Sponge like, Oil removal, Gamma-ray, Environmental social sciences, Chemistry, Materials science

## Abstract

The newest method for recycling waste linear low-density polyethylene (LLDPE) is the thermo-catalytic degradation technique known as catalytic pyrolysis. Typically, it is limited by 500–800 °C high temperatures. Catalytic pyrolysis releases toxins and forms harmful carbonized char. The current study is based on exposing wasted LLDPE to different gamma irradiation doses and then pyrolysis in castor oil (150–300 °C). The output product of Ir-(rLLDPE) is turned into another compound with a new structural architecture (sponge-like). SEM analysis confirms conversion, showing sponge-like spicules and layers. Ir-(rLLDPE) is sponge-like with a soft, malleable, absorbent texture. The DSC demonstrates altered thermal properties, with a melting point at 121 °C splitting into two peaks (endothermic at 117 °C and exothermic at 160 °C). The exothermic peaks signify the curing process of the sponge-like material. Ir-(rLLDPE) is assessed as an adsorbent for aqueous oils and solvents. The study examines irradiation doses, pyrolysis temperature, and time on adsorbent capacity. The oil removal obeys the Langmuir isotherm with monolayer adsorption, with a maximum adsorption capacity of 24.75 g/g of waste oil and 43 g/g of 1,1,2,2-tetrachloroethane. Squashing maintains adsorption after 20 reuses. Data shows sponges effectively clean marine oil spills and solvents.

## Introduction

Marine ecosystems are seriously threatened by oil spills, which pose significant economic losses and ecological harm^1–3^. With the growth of marine transportation and oil and gas development, the frequency of oil spills has increased, exacerbating the vulnerability of already fragile ecosystems^4–7^. Furthermore, various industries, including textiles, leather, and petrochemicals^8^, contribute to contaminating wastewater with different oils, which poses an environmental risk and has indirect links to climate change^9^. The consequences of oil spills, such as coral reef bleaching, hypothermia in nearby species, and even human health issues, highlight the urgent need for practical solutions^10,11^. Recycled materials, such as recycled plastics, metals, and paper, reduce the need for virgin resources and help divert waste from landfills^12^ by utilizing eco-friendly materials in various industries, such as packaging, construction, and biomedical applications^13,14^. Sustainable design and manufacturing practices prioritizing using eco-friendly materials contribute to a more circular economy by promoting resource efficiency and waste reduction^15^. Adsorption materials offer numerous advantages, including affordability, ease of usage, and environmental viability^16,17^. Traditional oil absorbents, such as silica^18^, graphite^19^, straw^20^, and polypropylene^21,22^, have been widely studied due to their practicality and cost-effectiveness.

Among the various methods for treating wastewater and oil pollution, physical adsorption techniques are crucial due to their simplicity and low cost^23^. These techniques are particularly effective for addressing oil spills^24^. Physical adsorption materials, including textiles, films, and sponges, possess porous structures that selectively absorb and separate oil and/or organic solvents while resisting water^25^. Commercially available sorption materials exhibit high capacities, mostly made of synthetic polymers like polyurethane (PU) and polypropylene (PP)^26–30^. Energy recovery plays a significant role in addressing the issue of waste materials^31^. Different chemical treatments and energy recovery technologies like gasification, hydrocracking, glycolysis, and pyrolysis can be employed^32–35^. Pyrolysis, in particular, is a versatile process that allows the thermal degradation of various organic and inorganic waste materials^36^. Pyrolysis can be conducted via a thermal (conventional) or catalytic route^37^. While the thermal route occurs without external agents, the catalytic route involves using an external agent to aid the pyrolysis process. Catalytic pyrolysis helps overcome the challenges associated with high-temperature demands, reaching up to 900 °C, and enables the optimization of process parameters and the production of higher-quality end products^38^.

The sustainability conditions of the current sponge-like materials are based on several items, such as (1) waste reduction and utilization: using waste LLDPE as a precursor material significantly reduces plastic waste in the environment^39^. By converting waste into functional adsorbents, our approach addresses the issue of plastic pollution while creating valuable products. (2) Resource efficiency: the synthesis of sponge-like materials capitalizes on the inherent properties of waste LLDPE. This approach minimizes the need for additional resources and energy, contributing to overall resource efficiency and minimizing the environmental footprint^40^. (3) Pollution mitigation^41^: the primary purpose of our sponge-like materials is the efficient removal of trace-level diesel oil from water. Our approach directly contributes to environmental protection and restoring polluted water bodies by effectively addressing oil pollution. (4) Renewable and low-cost feedstock^42^: LLDPE waste, as a renewable and readily available resource, is the basis for our materials. This approach offers a cost-effective and sustainable alternative to traditional adsorption materials. (5) Long-term reusability^43^: our study demonstrates the reusability of the sponge-like materials over multiple cycles, highlighting their long-term effectiveness and potential to reduce the need for frequent replacements. This aligns with sustainable practices by extending the lifespan of the adsorbent. (6) Potential for circular economy^44^: the transformation of waste LLDPE into valuable adsorbents contributes to the principles of a circular economy by closing the loop on plastic materials and creating new applications for discarded resources. In response to the above valuable items, we have achieved the sustainability approach of our new material to enhance the novelty and depth of our analysis, eliminate redundancy, and improve the reliability of results and descriptions throughout the manuscript.

The present study explores the production of Ir-(rLLDPE) sponge through gamma irradiation and catalytic thermal pyrolysis of waste LLDPE in castor oil. The unique properties of Ir-(rLLDPE) sponge, such as its high oil adsorption capacity, chemical stability, and ease of handling, make it a promising candidate for adsorbing organic pollutants. Irradiation induces structural modifications in the sponge, increasing the surface area and introducing new functional groups. These modifications significantly enhance the adsorption capacity and selectivity of the sponge towards various organic compounds. Understanding the adsorption kinetics, equilibrium behavior, and adsorption mechanism is crucial to optimizing the Ir-(rLLDPE) sponge design and application in oil spill remediation and water treatment. This study investigates the adsorption characteristics of Ir-(rLLDPE) sponge towards 1,1,2,2-tetrachloroethane, a representative organic solvent, under different radiation doses. The adsorption kinetics are evaluated using pseudo-first- and pseudo-second-order models, while the equilibrium adsorption capacity is analyzed using Langmuir and Freundlich isotherms. Furthermore, the effectiveness of Ir-(rLLDPE) sponge in removing diesel fuel from water is assessed through changes in UV–visible spectroscopy and visual observation of diesel-in-water emulsions before and after separation. Our study presents a pioneering approach that leverages recycled waste linear-low-density polyethylene (LLDPE) to synthesize a novel class of sponge-like materials with exceptional capabilities for adsorbing trace-level diesel oil from water. This innovative method transforms discarded waste into a valuable resource, demonstrating a sustainable and environmentally responsible solution for pollution control. The novelty of this study lies in the application of irradiated Ir-(rLLDPE) sponge as an efficient adsorbent for organic solvents and oil contaminants at trace level, the exploration of adsorption kinetics using advanced modeling techniques, and the comprehensive evaluation of adsorption performance using spectroscopic analysis and visual observation. These novel aspects contribute to advancing adsorbent materials and provide valuable insights for developing efficient oil spill remediation and water treatment strategies.

## Materials and methods

### Materials

Castor oil was supplied from Algomhoria Chemical Co. Cairo, Egypt. Oils such as crude oil 1 (light), crude oil 2 (medium), crude oil 3 (heavy), waste oil, motor oil, pump oil, and refrigeration oil (the oil properties are summarized in Table 1) and solvents such as cyclohexanone, petroleum ether, toluene, carbon tetrachloride, 1,2-dichloroethane, n-heptanone, methanol, butanone, benzen, 1,1,2,2-tetrachloroethane, methylene chloride, diethyl ether, tetrahydrofuran, and dimethylformamide were collected from the market and used as received.

**Table 1:** Physical properties of some of the tested oils.

Oil type	Density at 15 °C (g/mL)	Viscosity at 40 °C (cSt)	Flash point (°C)
Crude oil 1 (light)	0.819	2.4	-20
Crude oil 2 (medium)	0.853	5.2	35
Crude oil 3 (heavy)	0.920	42	90
Waste oil	0.895	100	220
Motor oil	0.880	65	190
Pump oil	0.910	750	250
Refrigeration oil	0.985	100	190

### Pre-irradiation process of recycled LLDPE by gamma rays ^60^Co-cell

The dose rate was applied at 0.9 kGy/h using Co-60 γ-cell-220 source at the National Center for Radiation Research and Technology (NCRRT), Egyptian Atomic Energy Authority (EAEA), Nasr City, Cairo, Egypt. The pre-irradiation process of recycled LLDPE (Liner Low-Density Polyethylene) using gamma rays from a ^60^Co-cell involves several steps to prepare the material before the irradiation process. (1) The first step is to collect and sort the recycled LLDPE material. This typically involves gathering post-consumer or post-industrial plastic waste and separating it based on its polymer type, color, and level of quality. (2) The collected recycled LLDPE is thoroughly cleaned and washed to remove impurities such as dirt, dust, residues, and organic contaminants. This step helps ensure the quality and purity of the material. (3) The cleaned recycled LLDPE is shredded into smaller pieces to increase the surface area and facilitate uniform irradiation. Size reduction can be done using mechanical methods like cutting, grinding, or pulverizing. (4) After the washing and drying step, the rLLDPE samples are carefully packaged to be read during the irradiation process. Suitable packaging materials, such as polyethylene bags, ensure the samples remain intact and shielded from external factors. Finally, the irradiation process setup is placed in a controlled environment near the ^60^Co-cell radiation source.

The rLLDPE was irradiated at different doses (0, 50, 75, and 100 kGy) in a solid state at ambient temperature. Various effects can occur, depending on the specific dose and the characteristics of the polymer such as. Irradiation doses in the 50–100 kGy range can induce crosslinking or degradation in rLLDPE. This process can improve the mechanical strength, chemical resistance, and thermal stability of the Ir-(rLLDPE), improving the material properties.

### Post-irradiation and thermal treatment conversion of Ir-(rLLDPE) to sponge-like material

The conversion process from Ir-(rLLDPE) to a sponge-like material involves additional steps after the irradiation process using heating in castor oil. (1) The irradiated rLLDPE samples, which have undergone the washing and drying steps mentioned earlier, are measured and weighed to obtain 0.5 g portions. (2) Each 0.5 g sample is immersed in 50 mL of castor oil. Castor oil is a vegetable oil that can serve as a plasticizer, softening the polymer and facilitating further processing or testing. (3) The samples are subjected to a temperature range of 150–300 °C. The specific temperatures within this range are determined based on the experimental requirements and the desired effects on the rLLDPE. (4) After the specified heating duration, the samples can cool to room temperature. Additional post-treatment steps may be performed depending on the experimental setup or subsequent analysis, such as washing and drying.

### Experimental setup for oil adsorption rate and uptake capacity assessment using Ir-(rLLDPE) sponge-like material

The rate of oil permeation into the pores of Ir-(rLLDPE) sponge-like material was investigated to assess its effectiveness in sorbing total crude oil spills from water media. The sorption performance was evaluated through a conventional sorption experiment, where the uptake capacities of the sponge-like sorbents were determined after various contact time intervals. The experimental setup is as follows: (1) preparation of crude oil–water mixture by adding 10 mL of crude oil to 100 mL of water and stirring for 10 min. (2) A constant weight of Ir-(rLLDPE) sponge-like sorbent, 0.1 g, was carefully placed onto the oil surface. The sorbent was allowed to float freely on the oil–water mixture. Contact intervals up to 60 min were used to study the sorption kinetics. (3) After each contact time interval, the oil-containing sorbents were removed from the mixture. The sorbents were left to drip for 30 s to allow excess oil to drain off. (4) The sorbents were weighed after dripping to assess their uptake capacities in g/g. The supplementary data represent the uptake capacity, kinetic studies, and isotherm model studies of oil removal using Ir-(rLLDPE) sponge-like.

### Characterization

The identification of microplastics was confirmed through DSC measurements using a PerkinElmer DSC 4000 instrument. Each extracted substance, weighing 3.0 mg, was analyzed in an aluminum crucible under a nitrogen atmosphere with a temperature range of 30–350 °C and a heating rate of 10 °C per minute. The surface morphology of the Ir-(rLLDPE) sponges was examined using a ZEISS EVO 15 microscope, and the elemental distribution was investigated through coupled SEM/EDX mapping analysis using a BRUKER Nano GmbH 410-M instrument.

### The proposed mechanism of Ir(rLLDPE) sponge formation

**Figure 1:**
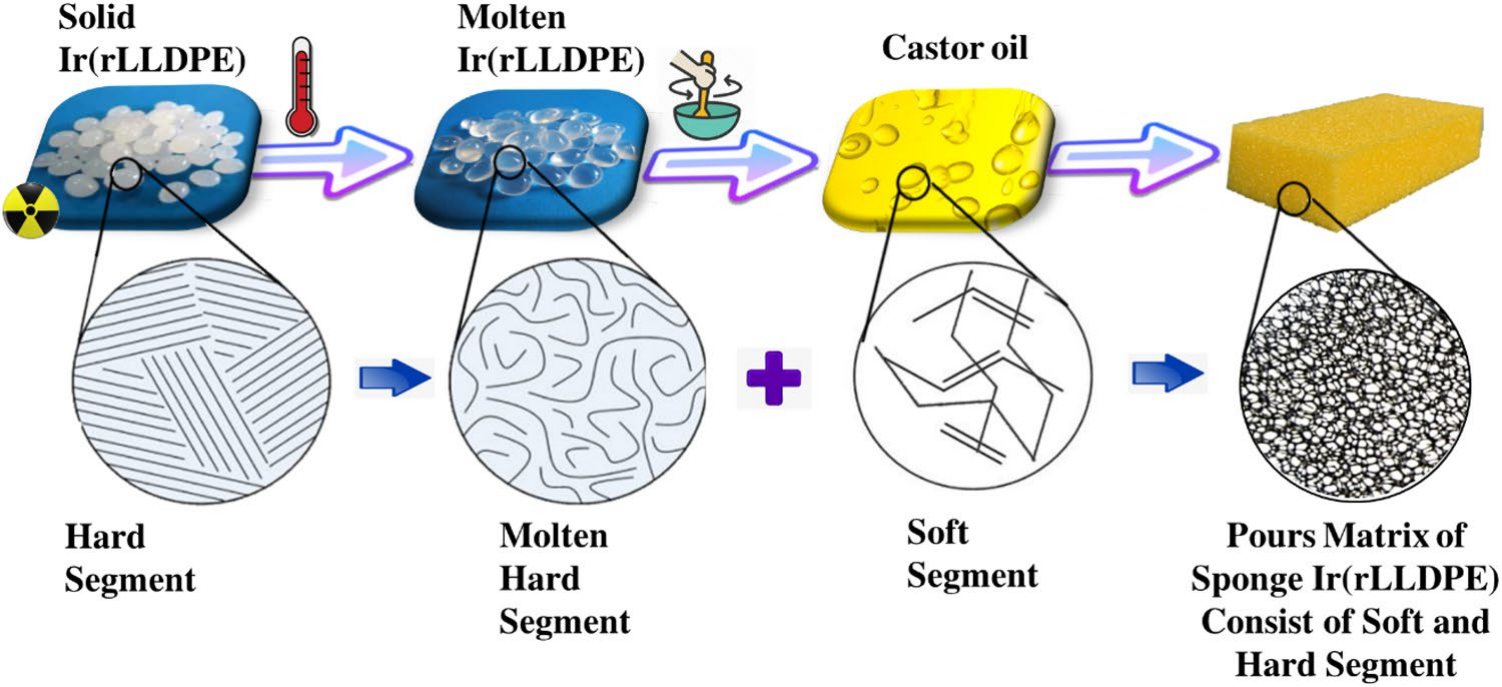
The synthesis process of the Ir(rLLDPE) sponge material. The diagram depicts the transformation from solid Ir(rLLDPE) with distinct hard segments. Upon heating in castor oil, the hard segments turn molten, and the addition of castor oil acts as a plasticizer, contributing to the soft segment. The resulting dual-phase material, with a combination of flexible and rigid elements, is structured in the form of a sponge.

Figure 1 provides a brief description of how sponge pours matrix of Ir(LLDPE) with distinct hard and soft segments, involving solid Ir(rLLDPE), molten Ir(rLLDPE) and castor oil. The start material is composed of Ir(rLLDPE) after being exposed to irradiation doses, which has distinct hard segments of –(CH_2_–CH_2_–CH_2_)–. The hard segment is in a molten state after the heating process in castor oil. The addition of castor oil to the composition indicates the presence of a plasticizer or a softening agent. Castor oil is known for its lubricating properties and can enhance the flexibility and elasticity of the material. This addition likely contributes to the soft segment of the polymer. The combination of soft (castor oil) and hard segments (molten Ir(rLLDPE) indicates a dual-phase material. This configuration is likely designed to achieve a balance of mechanical properties, combining the toughness and flexibility of the soft segment with the strength and rigidity of the hard segment. The material is structured in the form of a sponge, implying a porous and possibly lightweight configuration. The sponge is composed of both soft and hard segments, suggesting a combination of flexible and rigid elements within the structure.

## Results and discussion

### Structural analysis of irradiated rLLDPE using FTIR spectroscopy

**Figure 2:**
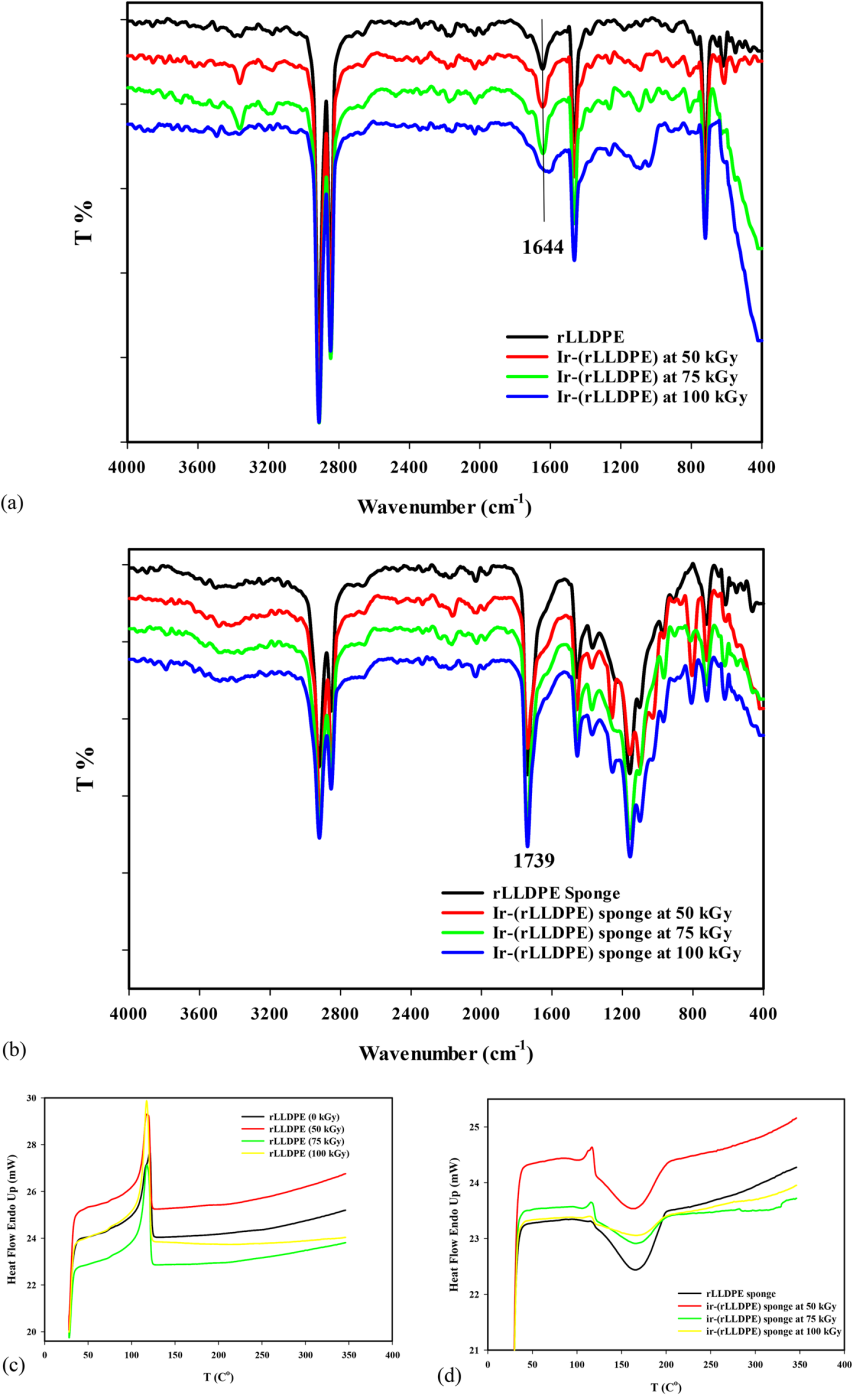

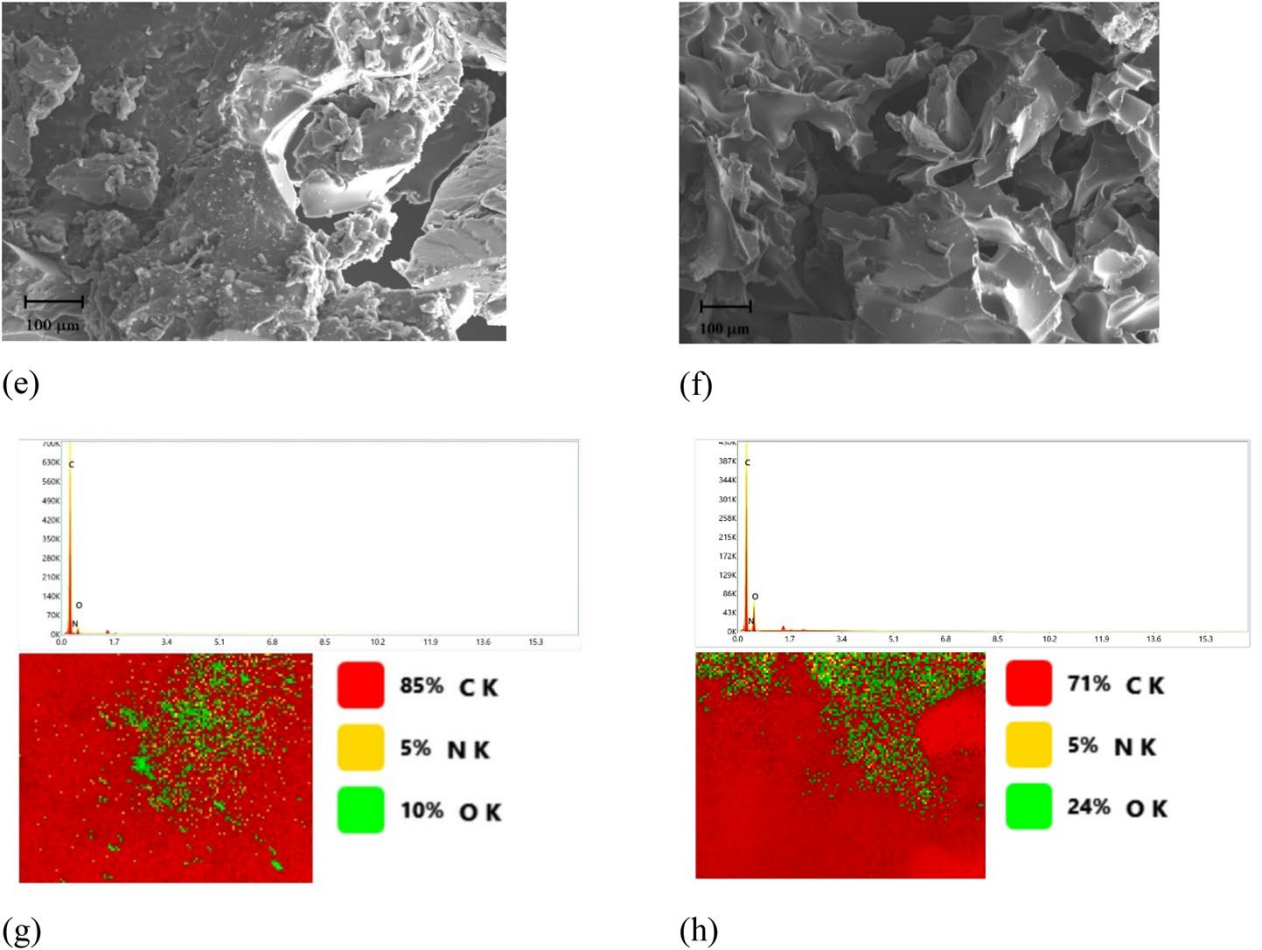
FTIR analysis of Ir-(rLLDPE) (**a**) and Ir-(rLLDPE) sponge like (**b**), the thermal DSC analysis of Ir-(rLLDPE) samples at different irradiation doses (**c**) in plastic form and (**d**) in sponge like form, the SEM image of (**e**) rLLDPE spong like and (**f**) the Ir(rLLDPE) sponge like, EDX/mapping analysis of (**g**) rLLDPE spong like and (**h**) the Ir(rLLDPE) sponge like.

The FTIR spectrum analysis investigated the structural changes in irradiated rLLDPE at various doses. The peaks observed in the FTIR spectra provide information about the vibrational modes of different functional groups present in the Ir-(rLLDPE) material. In Fig. 2a, the FTIR peaks observed at approximately 2912 cm^−1^ and 2848 cm^−1^ correspond to the asymmetric and symmetric stretching vibrations of CH_2_ groups, respectively^45^. These peaks indicate the presence of CH_2_ groups in the polymer structure. The peak at around 1461 cm^−1^ represents the bending vibrations of CH_2_ groups^46^. This peak confirms the presence of CH_2_ groups in the polymer chains. The peak at 718 cm^−1^ indicates the rocking vibration of CH_2_ groups. This peak provides further evidence of the presence of CH_2_ groups in the polymer structure. The 3380 cm^−1^ and 1644 cm^−1^ peaks are associated with the stretching vibrations of the OH and C=O bonds, respectively^47^. These peaks may arise from the presence of plasticizer additives in rLLDPE. The intensities of two peaks at 1644 cm^−1^ correspond to the stretching vibration of the C=O bond and at 3380 cm^−1^ correspond to the stretching vibration of the OH bond becoming low intensity and disappearing completely at a sample of Ir(rLLDPE) irradiated at 100 kGy due to the elimination and degradation of plasticizers molecules. The FTIR spectrum analysis in Fig. 2b was conducted to investigate the impact of castor oil on the formation of a sponge-like structure in a polymer material. The analysis revealed the presence of new peaks in the FTIR spectra, suggesting the involvement of castor oil in the structural transformation. These peaks are attributed to specific functional groups present in castor oil. A new peak appears at 1739 cm^−1^ in the FTIR spectrum, corresponding to the C=O stretching vibrations of the ester functional group^47^. This peak suggests the presence of ester bonds found in triglycerides composing castor oil. In addition, the FTIR peaks in the range of 1202–1074 cm^−1^ exhibit the stretching vibrations of the C–O bonds in the ester functional group. The appearance of this peak further confirms the presence of ester bonds in the sponge-like structure influenced by castor oil^48^. Finally, the FTIR spectrum analysis of Ir(rLLDPE) sponge-like samples after thermal treatment in castor oil reveals a new group of (C=O) in specific hydrophilic regions. These peaks correspond to the C=O stretching vibrations of the ester functional group and the stretching vibrations of the C–O bonds in the ester functional group. These observations provide evidence that the spongy-like structure formation is influenced by the presence of castor oil, which contains triglycerides with ester bonds. The interaction between the castor oil and the rLLDPE during the thermal treatment process contributes to the formation of hydrophilic /hydrophobic unique structure.

### The thermal DSC analysis of Ir-(rLLDPE) samples at different irradiation doses

Figure 2c and Table 2 represent the thermal DSC analysis performed on the Ir-(rLLDPE) samples at different irradiation doses (0, 50, 75, and 100 kGy). Table 2 represents the melting point (Tm), enthalpy change (ΔH), and area under the curve (mJ) for each sample. For the rLLDPE blank as received, the melting point (Tm) of the unirradiated rLLDPE sample is 121.03 °C. The enthalpy change (ΔH) for the melting transition is 129.95 J/g. The area under the curve, representing the total heat absorbed or released during the melting process, is 389.8 mJ. The sample irradiated at 50 kGy shows a slight decrease in the melting point (Tm)^49^ compared to rLLDPE, with a value of 117.26 °C. The melting transition's enthalpy change (ΔH) increases to 144.9 J/g. The area under the curve rises to 434.1 mJ. The sample irradiated at 75 kGy exhibits a similar melting point (Tm) of 117.7 °C compared to the 50 kGy sample. The melting transition's enthalpy change (ΔH) further increases to 147.7 J/g. The area under the curve slightly decreases to 413.16 mJ. The sample irradiated at 100 kGy maintains a similar melting point (Tm) of 117.3 °C compared to the previous samples. The melting transition's enthalpy change (ΔH) significantly increases to 183.41 J/g. The area under the curve further increases to 550.2 mJ. Finally, the thermal DSC analysis reveals changes in the melting behavior of the Ir-(rLLDPE) samples compared to the unirradiated rLLDPE. The irradiation process affects the melting point (Tm) and enthalpy change (ΔH)^50^ of the (LLDPE) samples. The increase in enthalpy change suggests changes in the crystallinity and thermal properties of the material due to irradiation. These observations indicate that the gamma irradiation process influences the thermal behavior and melting characteristics of the Ir-(rLLDPE) samples^51^. The irradiation process can indeed affect the melting point (Tm) and enthalpy change (ΔH) of LLDPE (linear low-density polyethylene) samples, especially in the case of recycled plastic materials that may contain plasticizer compounds. During irradiation, the plasticizer compounds in the recycled LLDPE can undergo radiolytic reactions^52^. These reactions can lead to the formation of radical groups, which can easily attach to the main chains of the polymer^53^. The attachment of these radical groups to the polymer chains can induce internal changes within the material^54^.

**Table 2:** Thermal DSC analysis of Ir-(rLLDPE) samples at different radiation doses.

Sample	Tm (°C)	ΔH (J/g)	Area (mJ)			
rLLDPE	121.03	129.95	389.8			
Ir-(rLLDPE) (50 kGy)	117.26	144.9	434.1			
Ir-(rLLDPE) (75 kGy)	117.7	147.7	413.16			
Ir-(rLLDPE) (100 kGy)	117.3	183.41	550.2			

Ir-(rLLDPE) Sponge like ( kGy)	Plastic form			Sponge form		
	Tm °C (endothermic)	ΔH (j/g)	Area (mJ)	Tc °C exothermic	ΔH (j/g)	Area (mJ)
0	–	–	–	166.3	− 92.83	278.51
50	117	7.99	23.9	165.4	− 67.06	201.19
75	116.6	3.44	10.3	166.2	− 43.26	129.80
100	115.6	1.79	5.3	168.2	− 27.31	81.90

The introduction of radical groups and internal changes can influence the crystallinity and thermal properties of the LLDPE. The crystalline structure of the polymer may be affected, leading to alterations in the melting point (Tm) and enthalpy change (ΔH) observed in the DSC analysis. The increased enthalpy change indicates changes in the energy required for the melting transition. This can be attributed to modifications in the crystallinity and thermal behavior induced by the radiolytic reactions and the attachment of radical groups. Examples of radical groups that can be formed during radiolytic responses in the presence of plasticizer compounds include alkyl radicals (such as methyl, ethyl, or butyl radicals), aryl radicals (such as phenyl or benzyl radicals), and acyl radicals (such as acetyl or benzoyl radicals)^55^.

These radical groups are highly reactive and can easily attach themselves to the polymer chains, leading to modifications in the structure and properties of the material. The attachment of alkyl radicals^56^, for instance, can result in branching or crosslinking of the polymer chains, affecting the crystallinity and thermal behavior of the material. It's important to note that the specific types of radical groups formed during radiolytic reactions can vary depending on the nature of the plasticizer compounds and the irradiation conditions^57^. The resulting radical groups can contribute to internal changes within the polymer matrix, influencing its properties, including melting point (Tm) and enthalpy change (ΔH)^58^.

Figure 2d and Table 2 display the DSC thermograms of Ir-(rLLDPE) sponge-like samples at different irradiation doses (0, 50, 75, and 100 kGy). As shown in the table, after the formation of the sponge-like structure, the DSC thermograms exhibit two distinct melting peaks, one endothermic and the other exothermic.

In the case of the unirradiated LLDPE sponge (0 kGy), only one exothermic curing peak is observed, and the endothermic melting peak of LLDPE is completely absent. For the irradiated Ir-(rLLDPE) sponge samples, the endothermic melting peaks are observed at temperatures ranging from 115.6 to 117 °C. These peaks correspond to the melting of the polymer within the sponge-like structure. The enthalpy (ΔH) was decreased and associated with ranges from 1.79 to 7.99 J/g, indicating a decrease in the amount of remaining LLDPE molecules that did not convert to sponge structure due to the per irradiation process induced crosslinking reaction of rLLDPE.

As shown in Table 2, the enthalpy (ΔH) values of the Ir-(rLLDPE) sponge-like structure at doses of 50, 75, and 100 kGy are 7.99 J/g, 3.44 J/g, and 1.79 J/g, respectively. These values indicate a decrease in the enthalpy change with increasing irradiation dose. The enthalpy change during a thermal transition represents the heat the rLLDPE material absorbs. In this case, the enthalpy change corresponds to the remaining rLLDPE that does not convert to the sponge-like structure.

The decrease in enthalpy with increasing irradiation doses, particularly at 75 and 100 kGy, suggests that different reactions occur in the rLLDPE material. At a dose of 50 kGy, the crosslinking reaction becomes prominent. Crosslinking involves the formation of covalent bonds between polymer chains, resulting in a three-dimensional network structure. This crosslinking reaction restricts the mobility of polymer chains and needs high energy to melt, increasing the enthalpy value during the melting transition. Crosslinks prevent the material from fully transitioning to molten, resulting in a greater enthalpy change. The chain scission reaction becomes dominant at doses of 75 and 100 kGy. Chain scission refers to the breaking of polymer chains, resulting in shorter chain lengths and the formation of free radicals. The chain scission reaction leads to a reduction in the overall crystallinity and molecular weight of the material. As a result, the enthalpy change during the melting transition is further decreased. The combination of crosslinking and chain scission reactions at different irradiation doses leads to changes in the thermal behavior and properties of the rLLDPE material. The observed decrease in enthalpy with higher irradiation doses indicates the occurrence of scission reactions. The exothermic peaks in the thermograms represent the curing process (T_c_) of sponge and occur at temperatures ranging from 165.4 to 168.2 °C. These exothermic peaks' negative enthalpy change values (ΔH) indicate heat released during curing.

The exothermic peaks observed in the thermograms of the Ir-(rLLDPE) sponge-like samples represent the curing process of the sponge. During the curing process, chemical reactions occur within the material, leading to the formation of a crosslinked network and the consolidation of the sponge structure.

The exothermic nature of these peaks indicates that heat is released during the curing process. The negative enthalpy change values (ΔH) associated with these exothermic peaks further confirm the heat release. Negative ΔH values indicate that heat is evolved or released from the system during curing. This heat release can be attributed to the exothermic nature of the cross-linking chemical reactions. The crosslinking reactions generate heat as bonds are formed, resulting in the observed exothermic peaks and negative ΔH values. Therefore, exothermic peaks and negative enthalpy change values in the thermograms of the Ir-(rLLDPE) sponge-like samples indicate a curing process similar to what is observed in the DSC thermograms of polyurethane sponge^59^. The DSC thermograms and the data in Table 1 demonstrate the altered melting and curing behavior of Ir-(rLLDPE) sponge-like samples compared to unirradiated LLDPE. The irradiation process induces the formation of a new thermal profile characterized by two distinct melting peaks and modified enthalpy values, indicating changes in the material's crystalline structure and thermal properties.

### EDX analysis and morphological characterization of rLLDPE and Ir-(rLLDPE) sponge-like samples

Figure 2e and f show SEM images of the (rLLDPE) and Ir-(rLLDPE) sponge-like structures, respectively. The images reveal that the sample has a condensed structure with increased porosity in the irradiated samples compared to the non-irradiated samples. The increased porosity suggests that the irradiation process has influenced the morphology of the sponge-like structure, resulting in a more porous and open structure. This increased porosity can benefit applications such as oil absorption, as it provides a larger surface area and improves accessibility for interaction with the surrounding environment. Figure 2g and h represent the EDX/mapping curves of both rLLDPE and Ir-(rLLDPE) sponge-like samples. The EDX analysis provides information about the elemental composition of the materials. In the EDX curves, three elements are observed: carbon (C), nitrogen (N), and oxygen (O). The presence of carbon is expected as it is a significant component of the polymer matrix. The detection of nitrogen and oxygen atoms in EDX analysis suggests the presence of additional compounds in the recycled LLDPE samples, such as plasticizers and UV protection, antioxidants, thermal stabilizers and slip compounds^60–62^. In the case of the Ir-(rLLDPE) sponge-like sample, the amount of (O) is increased from 10 to 24% after thermal treatment in castor oils. The increase in the percentage of oxygen (O) after thermal treatment in castor oil indicates a change in the composition of the Ir-(rLLDPE) sponge-like material. The presence of the (C=O) groups, which are attached to the rLLDPE sponge-like structure, contributes to the increased percentage of oxygen. Introducing (C=O) groups through the thermal treatment in castor oil imparts hydrophilic properties to the material. The presence of oxygen, particularly in the form of (C=O) groups, enhances the materials affinity for water and increases its hydrophilicity. This can be beneficial in oil separation applications.

### Effect of temperature, duration and gamma irradiation dose on sponge like formation and adsorption capacity of Ir-(rLLDPE)

**Figure 3:**
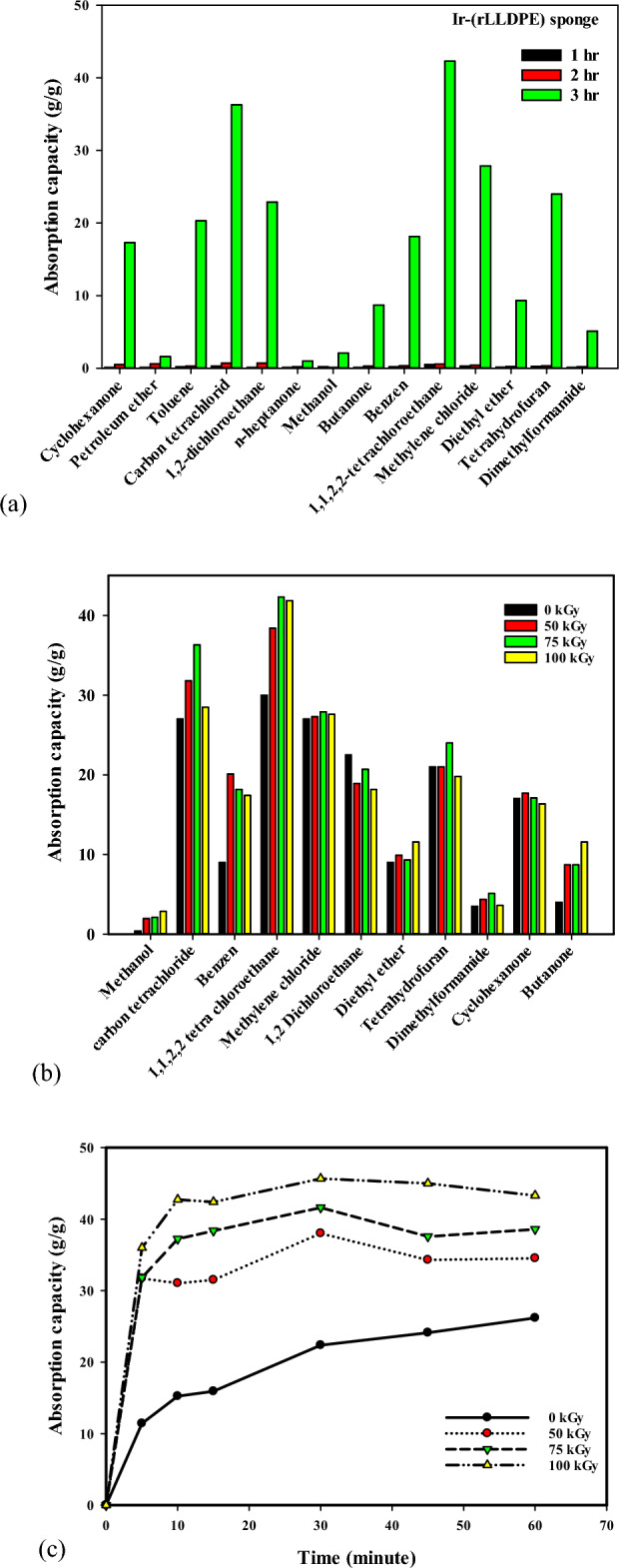
Represent the maximum adsorption capacities of Ir-(rLLDPE) sponge prepared at different durations of pyrolysis against 14 organic solvents (**a**), the maximum adsorption capacities of Ir-(rLLDPE) sponge prepared at different gamma irradiation doses (0, 50, 75 and 100 kGy) against 11 organic solvents (**b**), and (**c**) show the adsorption rate of 1,1,2,2-tetrachloroethane by Ir-(rLLDPE) sponge irradiated at different doses.

The preparation of the Ir-(rLLDPE) sponge involved exposing irradiated samples at 75 kGy to temperatures ranging from 150 to 300 °C. Among the tested temperatures, it was found that the desired sponge shape was only achieved at 300 °C. This indicates that the thermal transformation of the irradiated rLLDPE into a sponge-like structure required a specific temperature, and 300 °C provided the most favorable conditions. The Ir-(rLLDPE) sponge was evaluated for its adsorption performance against 14 types of organic solvents, and the results in Fig. 3a indicate the maximum amount of each solvent that can be adsorbed by the Ir-(rLLDPE) sponge.

Additionally, the adsorption capacity of a solvent on the Ir-(rLLDPE) sponge can depend on its chemical structure and polarity. Solvents with different chemical structures and polarities interact differently with the Ir-(rLLDPE) sponge, leading to variations in their adsorption capacities. For example, solvents with polar molecules, such as cyclohexanone (C=O) and tetrahydrofuran (–O–) tend to have higher adsorption capacities. This is because polar solvents can form more robust interactions with the functional groups in the Ir-(rLLDPE) sponge, such as hydrogen bonding or dipole–dipole interactions. These interactions facilitate the adhesion and retention of the solvent molecules within the sponge structure. Halogen solvents containing specific functional groups (Cl), like carbon tetrachloride, 1,2-dichloroethane, and methylene chloride, can exhibit higher adsorption capacities. The presence of halogen atoms in these solvents can promote stronger interactions with the Ir-(rLLDPE) sponge due to the possibility of halogen bonding or other specific interactions with the sponge material. The polarity of solvents can also influence adsorption capacity. Solvents with more significant nonpolar properties, like toluene and benzene, may have increased adsorption capacities due to hydrophobic-hydrophobic interactions. Solvents with more significant polar properties, such as methanol and dimethyl formamide, may have low adsorption capacities. Finally, a solvent's chemical structure and polarity are crucial in determining its adsorption capacity on the Ir-(rLLDPE) sponge. By understanding these factors, it becomes possible to select solvents with desirable properties for specific applications, optimizing the performance of the sponge as an effective adsorbent.

Moreover, Ir-(rLLDPE) sponge-like samples are also tested with other organic solvents irradiated at different irradiation doses, as shown in Fig. 3b**.** It can be observed that the Ir-(rLLDPE) sponge-like samples, particularly the 75 kGy irradiated sample, exhibit high adsorption capacities for various organic solvents. The maximum adsorption capacities are achieved for 1,1,2,2-tetrachloroethane and carbon tetrachloride, with values of 43 g/g and 35 g/g, respectively. Methylene chloride, tetrahydrofuran, and benzene also show significant adsorption capacities, with 25 g/g, 23 g/g, and 18 g/g, respectively.

However, the adsorption capacities for methanol and dimethylformamide are comparatively lower, with values of 3 g/g and 5 g/g, respectively. All the irradiated samples, including the 75 kGy irradiated sample, demonstrate superior adsorption capacities compared to the unirradiated rLLDPE sponge. The increased adsorption capacities of the irradiated samples can be attributed to the structural modifications induced by the irradiation process. These modifications may enhance the sponge material's surface area, porosity, and chemical reactivity, improving adsorption performance for a wide range of organic solvents. Overall, the results suggest that the irradiated Ir-(rLLDPE) sponge-like samples have great potential as effective adsorbents for various organic solvents, offering a promising solution for environmental remediation and pollution control applications. To understand what happened, it is essential to classify it based on solvent polarity.

The solvents can be classified according to their adsorption capacity as follows: high adsorption capacity: 1,1,2,2-tetrachloroethane (42.29 g/g), carbon tetrachloride (36.29 g/g), methylene chloride (27.90 g/g), tetrahydrofuran (24 g/g), moderate adsorption capacity such as 5. Benzene (18.15 g/g); 1,2-dichloroethane (20.70 g/g); cyclohexanone (17.10 g/g); and low adsorption capacity such as 8. Diethyl ether (9.30 g/g); butanone (8.69 g/g). dimethylformamide (5.09 g/g); methanol (2.1 g/g). Also, solvents such as 1,1,2,2-tetrachloroethane, carbon tetrachloride, methylene chloride, and tetrahydrofuran exhibit high adsorption capacities. These solvents have a strong affinity for the Ir-(rLLDPE) sponge material, allowing them to be absorbed in large quantities. They are likely non-polar or have low polarity, which facilitates their interaction with the hydrophobic surface of the sponge. Solvents like benzene, 1,2-dichloroethane, and cyclohexanone have moderate adsorption capacities. These solvents may have a slightly lower affinity for the sponge material than the solvents with high adsorption capacity. They could have a slightly higher polarity or different molecular structures affecting their sponge interaction. Solvents such as diethyl ether, butanone, dimethylformamide, and methanol exhibit lower adsorption capacities. These solvents may have higher polarity or different chemical structures that limit their interaction with the hydrophobic surface of the sponge. As a result, they are absorbed in lower quantities than the other solvents. The classification of solvents based on their adsorption capacity provides insights into the compatibility and effectiveness of the Ir-(rLLDPE) sponge material for different solvents. Solvents with higher adsorption capacities can be preferred when maximum oil adsorption is desired, while solvents with lower adsorption capacities may be suitable for specific applications or requirements.

Figure 3c shows the adsorption capacity of 1,1,2,2-tetrachloroethane with time (minutes) by Ir-(rLLDPE) sponge irradiated at doses of (0, 50, 75, and 100 kGy). The adsorption capacity of 1,1,2,2-tetrachloroethane generally increases with time for all irradiation doses. Additionally, it can be observed that the adsorption capacities of the irradiated sponge samples (50, 75, and 100 kGy) are higher compared to the unirradiated sponge (0 kGy) at most time points. This indicates that irradiation enhances the adsorption capacity of the Ir-(rLLDPE) sponge for 1,1,2,2-tetrachloroethane. The adsorption capacity of 1,1,2,2 tetrachloroethane significantly increased with irradiation dose, with percentage changes ranging higher approximately 68.82–214.84% for 100 kGy compared to the blank sample at 0 kGy. Indicating the superior adsorption capacity achieved at 100 kGy.

### Evaluate the oil adsorption capacity of Ir-(rLLDPE) sponge obtained via pyrolysis of pre-irradiated (rLLDPE) samples

**Figure 4:**
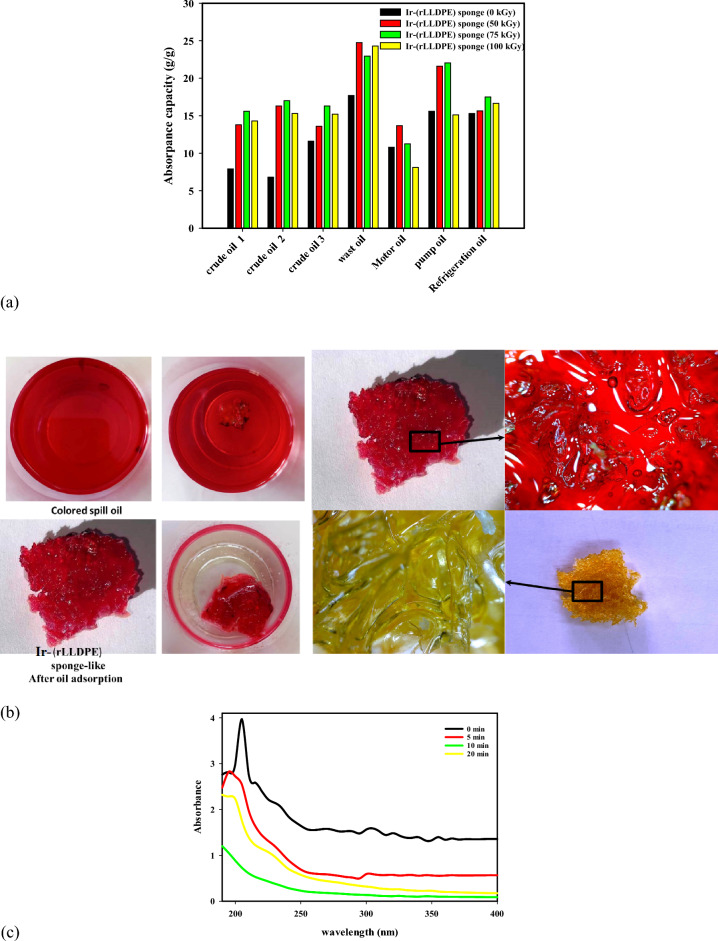

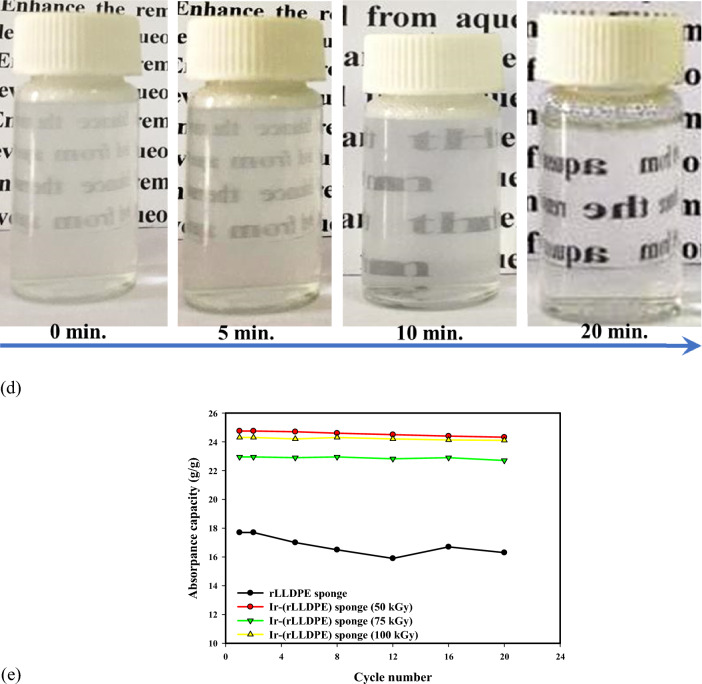
(**a**) The oil adsorption capacities of the sponge at different irradiation doses (0, 50, 75, and 100 kGy), (**b**) the red-colored oil spill in water was efficiently removed by using Ir(rLLDPE) sponge-like, (**c**) show the UV vis spectrophotometer of diesel-in-water emulsions, (**d**) the photos of diesel-in-water emulsions before and after separation using Ir-(rLLDPE), after (0, 5, 10, and 20 min), and (**e**) shows the reusability of rLLDPE and Ir-(rLLDPE) sponges at different radiation doses for waste oil absorption.

The pre-irradiated samples (0, 50, 75, and 100 kGy) of (rLLDPE) were pyrolyzed in castor oil at 300 °C for 3 h. The oil adsorption capacity of Ir-(rLLDPE) sponge, obtained through the pyrolysis of pre-irradiated (rLLDPE) samples, was evaluated against seven different oils. Figure 4a represents the oil adsorption capacities of the sponge at different irradiation doses (0, 50, 75, and 100 kGy). The results indicate that the adsorption capacity varies depending on the type of oil and irradiation dose. Generally, higher irradiation doses correspond to increased adsorption capacities, suggesting that the modified sponge material is more effective in adsorbing and removing oils from the environment. Figure 4a percentage change compared to 0 kGy indicates that the adsorption capacity of the Ir-(rLLDPE) sponge for different oils varies with the irradiation dose. For crude oil 1, there is an increase in adsorption capacity with increasing irradiation dose, reaching the highest percentage change at 75 kGy (97.47%). However, the adsorption capacity slightly decreases at 100 kGy (81.01%) compared to 75 kGy. For crude oil 2, there is a significant increase in adsorption capacity with increasing irradiation dose, with the highest percentage change observed at 50 kGy (139.71%). The adsorption capacity continues to increase at 75 kGy (150.00%) and remains relatively high at 100 kGy (125.00%). For crude oil 3, the adsorption capacity shows a modest increase with irradiation dose, with the highest percentage change observed at 75 kGy (40.52%). The adsorption capacity at 50 kGy (17.24%) and 100 kGy (31.03%) is relatively lower. For waste oil, the adsorption capacity increases with irradiation dose, with the highest percentage change observed at 50 kGy (39.83%). The adsorption capacity slightly decreases at 75 kGy (29.62%) but remains relatively high at 100 kGy (37.29%). For motor oil, the adsorption capacity initially increases at 50 kGy (26.67%) but decreases at 75 kGy (4.17%) and 100 kGy (− 25.00%), indicating a decline in effectiveness at higher irradiation doses. For pump oil, there is an increase in adsorption capacity at 50 kGy (38.46%) and 75 kGy (41.67%), but a slight decrease at 100 kGy (− 3.08%). For refrigeration oil, there is a minimal increase in adsorption capacity at 50 kGy (2.29%).

The results suggest that the adsorption capacity of the Ir-(rLLDPE) sponge is influenced by the oil type and the irradiation dose. While higher irradiation doses generally improve the adsorption capacity, there may be an optimal dose range for each oil type beyond which effectiveness decreases. The variation in adsorption capacity with different irradiation doses can be attributed to several factors, such as. The irradiation process produces new C=O (carbonyl) groups from radiolytic plasticizer molecules or castor oil in the Ir-(rLLDPE) sponge-like samples. These functional groups can enhance the affinity of the sponge material towards oil molecules, promoting higher adsorption capacities. The presence of these C=O groups increases the interaction between the sponge and oil, leading to improved absorption. These changes can alter the physical properties of the sponge material, including its hydrophobicity and wettability.

The irradiation process may also affect the surface area and porosity of the sponge material. Higher irradiation doses can increase surface roughness^63^, enhancing the surface area for oil adsorption. Additionally, the formation of pores and cavities within the sponge structure can provide more sites for capturing oil molecules, further increasing the adsorption capacity. Irradiation can induce changes in the polymer structure, such as cross-linking or chain scission^64^. Irradiation can cause changes in the molecular weight of the polymer^65^. These changes can influence the mechanical strength and flexibility of the sponge material, which in turn affects its ability to absorb oils. An optimal molecular weight range may be achieved at certain irradiation doses, enhancing adsorption capacity. It is important to note that the effects of irradiation on the adsorption capacity can vary depending on the type of oil. Different oils have different molecular structures and chemical properties, which can interact differently with the modified sponge material. This is why we observe variations in the adsorption capacity among different oils at the same irradiation dose.

The increase in oil adsorption capacity with irradiation dose can be attributed to the formation of new C=O groups in the irradiated Ir-(rLLDPE) sponge-like samples. These C=O groups may enhance the affinity of the sponge material towards oil molecules, leading to improved oil adsorption capabilities. The presence of these functional groups can enhance the interaction between the sponge and oil, promoting higher adsorption capacities. This increase in oil adsorption capacity with irradiation dose suggests that the modified sponge material is more effective in removing and absorbing oils from the surrounding environment. Figure 4b shows the efficient removal of a red-colored oil spill in water using Ir(rLLDPE) sponge-like material. Unfortunately, no specific search results provide information on using Ir(rLLDPE) sponge-like material to remove oil spills in water. However, various methods can be used to clean up oil spills in water, such as chemical dispersion, burning, shoreline cleaners, biodegradation agents, manual removal, and mechanical removal. Using Ir(rLLDPE) sponge-like material to remove oil spills in water could be a promising method to protect the environment.

### UV–VISIBLE spectroscopy and reusability analysis of Ir-(rLLDPE) sponge for trace level diesel fuel adsorption

Figure 4c,d show UV–visible spectroscopy of the trace level of diesel fuel (50 mg/L) in a water/surfactant mixture adsorbed by Ir-(rLLDPE) sponge-like sample after various time intervals (0, 5, 10, and 20 min).

In this study, the adsorption of the trace level of diesel fuel was examined over different time intervals: 0, 5, 10, and 20 min. By analyzing the concentration of diesel fuel in the water/surfactant mixture before and after each time interval, we could observe any changes in the adsorption behavior.

The UV–visible spectroscopy analysis reveals the effective adsorption of diesel fuel by the Ir-(rLLDPE) sponge. The decrease in peak intensity at 210 nm with increasing adsorption time demonstrates the sponge's ability to rapidly remove diesel fuel from the solution. Notably, a gradual reduction in the peak's intensity is observed as adsorption time increases, culminating in complete removal after 20 min. This rapid and efficient adsorption process underscores the Ir-(rLLDPE) sponge's prowess as an oil adsorbent, particularly for trace-level diesel fuel removal. The study's results may reveal important information about the adsorption kinetics of diesel fuel at trace levels. For instance, the concentration of diesel fuel adsorbed onto the sample may increase with time, indicating a progressive adsorption process. Alternatively, the adsorption rate might plateau after a certain period, suggesting equilibrium between the diesel fuel concentration in the water/surfactant mixture and the amount adsorbed onto the sample. Understanding the behavior of trace levels of diesel fuel in water/surfactant mixtures is crucial for various applications, such as environmental monitoring, oil spill remediation, and water purification. By studying the adsorption characteristics, researchers can develop strategies to efficiently remove diesel fuel from contaminated water sources, contributing to protecting the environment and human health. Also, supplementary Video S1 demonstrates the rapid removal of crude oil using LLDPE sponge-like materials.

The reusability evaluation of the Ir-(rLLDPE) sponge compared to the rLLDPE sponge is displayed in Fig. 4e, which illustrates the superior reusability of the Ir-(rLLDPE) sponge in contrast to the rLLDPE sponge. The Ir-(rLLDPE) sponge maintains a consistently high adsorption capacity even after 20 cycles of adsorption and desorption. In contrast, the adsorption capacity of the rLLDPE sponge exhibits instability, leading to a significant decrease over multiple cycles. This exceptional reusability of the Ir-(rLLDPE) sponge can be attributed to the robust, attractive forces between the linear polyethylene bonds, ensuring structural integrity and stable performance throughout repeated cycles. Conversely, the rLLDPE sponge may experience structural degradation or a decline in active sites, causing diminished adsorption performance over time.

The demonstrated ability of the Ir-(rLLDPE) sponge to retain its adsorption capacity across numerous cycles underscores its practical potential. This enhanced reusability contributes to its cost-effectiveness and sustainability, minimizing the need for frequent replacement or disposal. These findings highlight the Ir-(rLLDPE) Sponge's promise as a dependable adsorbent for various environmental remediation and pollution control applications. The adsorption capacity of polyethylene is comparable to or better than that reported for other adsorbents listed in Table 3.

**Table 3:** Adsorption capacities of different adsorbents poyethylen.

Type of adsorbent	Adsorbed	Capacity	References
Recycled low-density polyethylene (LDPE) powder grafted by the cationic polymer	Crude oil	41 mg/g	^66^
HDPE aerogel coated natural rubber latex foam	Carbon tetrachloride	14 g/g	^67^
Poly(ethylene-co-vinyl acetate)	Toluene	27.39 g/g	^68^
GO/dodecylamine/polyethylene	Tetrahydrofuran	78 g/g	^69^
HDPE kitchen sponge	Chloroform	90 g/g	^70^
Polyethylene-blended polystyrene nanofibrous	Crude oil	119 g/g	^71^
Polyethylene over magnetite-multiwalled carbon nanotubes	Kerosene	3560 mg/g	^72^
Ir-(rLLDPE) sponge	1,1,2,2-Tetrachloroethane	43 g/g	Present work

### The adsorption kinetic study

**Figure 5:**
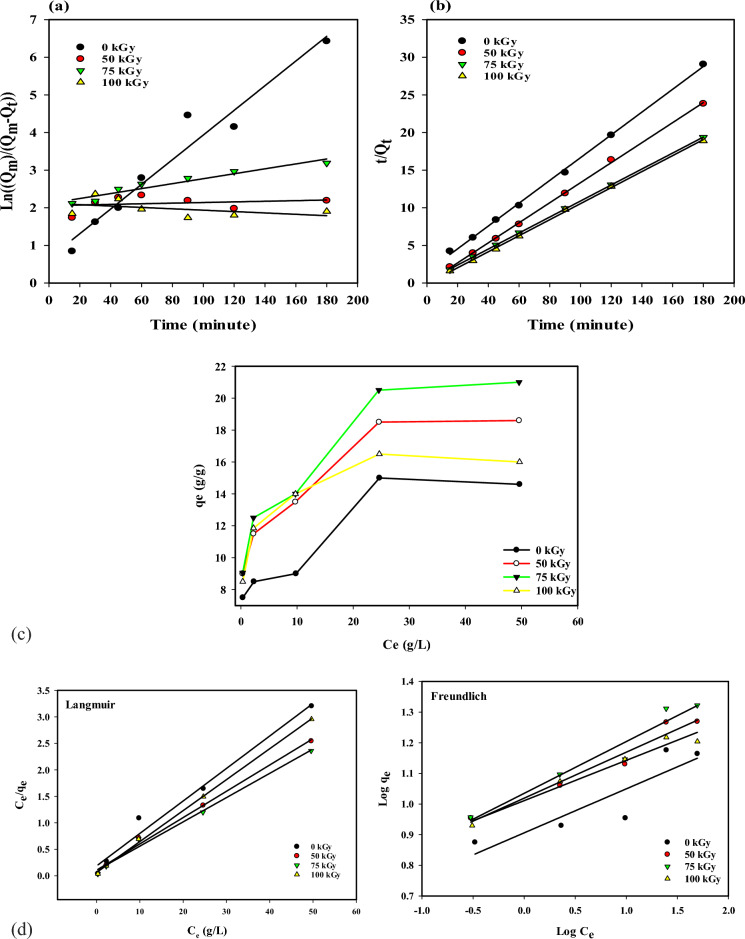
The adsorption kinetic with pseudo-first-order adsorption linear fitting (**a**) and Pseudo second-order adsorption linear fitting (**b**) of the Ir-(rLLDPE) sponges prepared at different irradiation doses (0, 50, 75, and 100 kGy), and (**c**,**d**) show the Langmuir and Freundlich adsorption isotherm models for the adsorption of Crude oil by Ir-(rLLDPE) sponges prepared at different irradiation doses (0, 50, 75, and 100 kGy).

The adsorption kinetics of 1,1,2,2 tetrachloroethane on Ir-(rLLDPE) sponge samples irradiated at doses of (0, 50, 75, and 100 kGy) are presented in Fig. 5a,b using pseudo-first and second-order models. The experimental adsorption kinetics data is conducted on the pseudo-first- and pseudo-second-order models. It is noted in Table 4 that the correlation coefficient R^2^ in the pseudo-second-order model is greater than that in the pseudo-first-order model for all samples at different irradiation doses.

**Table 4:** The kinetic parameters of pseudo-first-order and pseudo-second-order.

Samples Ir-(rLLDPE) sponge	Pseudo first-order		Pseudo second-order	
	K1	R^2^	K2	R^2^
(0 kGy)	0.032	0.9525	0.017	0.9987
(50 kGy)	0.0008	0.0558	0.940	0.9993
(75 kGy)	0.0065	0.9241	0.041	0.9999
(100 kGy)	− 0.0018	0.2015	0.040	0.9991

In the pseudo-first-order model, the rate constant (K1) and the correlation coefficient (R^2^) are reported in Table 4. The R^2^ values range from 0.0558 to 0.9525 for the different samples. The pseudo-first-order model assumes that the adsorption rate is directly proportional to the concentration difference between the sponge and the solvent, and the process follows first-order kinetics. In the pseudo-second-order model, the rate constant (K2) and the correlation coefficient (R^2^) are provided in Table 4. The R^2^ values for the pseudo-second-order model are consistently higher, ranging from 0.9987 to 0.9999 for the different samples. The pseudo-second-order model suggests that the solvent and sponge surface interactions influence adsorption. The adsorption rate depends on the concentration difference and the square of the solvent concentration.

Based on the higher R^2^ values obtained with the pseudo-second-order model, it can be concluded that it better fits the experimental adsorption kinetics data for all samples at different irradiation doses. Therefore, the pseudo-second-order model is considered more accurate in predicting the adsorption behavior of the Ir-(rLLDPE) sponge regarding solvent and oil absorption. These results indicate that the adsorption of 1,1,2,2-tetrachloroethane on the irradiated Ir-(rLLDPE) sponge is a complex process influenced by the concentration difference and the interaction between the solvent and the sponge surface. The pseudo-second-order model provides a more comprehensive understanding of the adsorption kinetics and can be used to predict the adsorption behavior of the sponge material in practical applications.

### The adsorption isothermal study

The adsorption isotherm illustrated in Fig. 5c,d shows the relationship between the quantity of oil adsorbate removed from the liquid phase and the mass of the Ir-(rLLDPE) per unit of temperature. The presence of adsorption isotherms is essential for designing efficient adsorption systems. A precise mathematical depiction of the equilibrium adsorption capacity is crucial for accurately predicting adsorption parameters and quantitatively comparing the adsorption behavior of different systems. The equilibrium parameters offer valuable insights into the adsorbent's sorption mechanism, surface properties, and affinity. Thus, it is necessary to determine the most suitable correlation for the equilibrium curves to optimize the design conditions of adsorption systems. This study employed Langmuir and Freundlich isotherms to analyze the equilibrium data^73^ The findings presented in Table 5 indicate that the equilibrium adsorption capacity of Ir-(rLLDPE) sponge samples irradiated at different doses (0 kGy, 50 kGy, 75 kGy, and 100 kGy) was analyzed using Langmuir and Freundlich, isotherm models. The results in Table 5 demonstrate that the Langmuir isotherm model better fits the equilibrium data, as indicated by higher R^2^ values than the Freundlich model. The maximum adsorption capacity (q_max_) increases with an irradiation dose up to 75 kGy and then slightly decreases at 100 kGy, suggesting an optimal irradiation dose for enhancing adsorption performance. The Langmuir isotherm assumes a monolayer adsorption process where adsorption occurs on a homogeneous surface with a limited number of active sites. It assumes that there is no interaction between adsorbed molecules and that the adsorption capacity reaches a maximum value (qmax) when the surface is fully covered with an adsorbate.

**Table 5:** Comparisons between Freundlich and Langmuir adsorption isotherm constants for Ir-(rLLDPE) sponge at different radiation doses.

Isotherm models	0 kGy	50 kGy	75 kGy	100 kGy
Langmuir				
q_max_ (g/g)	16.39	20.16	21.83	17.12
K_L_	0.327	0.47	0.455	0.94
R_l_	0.91	0.87	0.88	0.77
R^2^	0.9806	0.9937	0.9915	0.9989
Freundlich				
N	6.99	6.71	5.95	7.63
R^2^	0.793	0.956	0.947	0.964
K_f_	8.838	12.27	10.76	10.26

On the other hand, the Freundlich isotherm is an empirical model that describes adsorption on heterogeneous surfaces. It assumes that the adsorption capacity increases continuously with the adsorbate concentration, without a maximum value. It also considers multiple adsorption layers and assumes that adsorption occurs on different sites with different affinities. The better fitting of the Langmuir model in this case suggests that the adsorption process on the Ir-(rLLDPE) sponge samples follows a monolayer adsorption mechanism and that the surface is relatively homogeneous. It indicates that the adsorption capacity reaches a maximum value at a specific adsorbate concentration, consistent with the assumptions of the Langmuir model.

### Potential for scale-up and cost analysis

The potential for scaling up the production of Ir-rLLDPE sponge material presents a promising avenue for large-scale manufacturing. The synthesis process involves straightforward steps, primarily irradiation and thermal treatment, making it well-suited for adaptation to industrial-scale production. Below are some key points that underscore the feasibility and benefits of scaling up the production of Ir-rLLDPE sponge material. The Ir-rLLDPE sponge synthesis relies on simple and well-established processes such as irradiation and thermal treatment. These processes can be seamlessly integrated into industrial equipment. Larger irradiation chambers and conveyor systems can facilitate continuous irradiation of rLLDPE feedstock, allowing for efficient and high-volume production. Raw materials crucial for the synthesis, including waste LLDPE and castor oil, are abundantly available cheaply. This availability contributes to the economic feasibility of large-scale production. The reliance on cost-effective and readily available materials enhances the sustainability and economic viability of the entire manufacturing process. The fabrication procedure for the sponge does not involve intricate synthesis steps or require expensive reagents or catalysts. This simplicity is advantageous for straightforward and cost-effective scale-up. The absence of complex synthesis steps minimizes the risk of process bottlenecks, reducing the likelihood of complications during large-scale production. Utilizing the design of experiments (DOE) methodology allows for systematic process optimization. Identifying ideal parameters such as irradiation dose, temperature, and time becomes crucial for maximizing sponge yield and maintaining quality on an industrial scale. Process optimization ensures production operates at peak efficiency, enhancing productivity and minimizing resource wastage.

The produced sponge can be shaped into various forms, including blocks, sheets, balls, etc. This versatility in shaping allows for diverse applications across different industries. Industrial molding and extrusion equipment can be employed to mass-produce these shaped sponges efficiently, meeting the requirements of various applications. In conclusion, the potential for scale-up and cost analysis of producing Ir-rLLDPE sponge material on a large scale appears promising. Leveraging the simplicity of the synthesis process, the abundance of cost-effective raw materials, and the adaptability to industrial equipment, the manufacturing of Ir-rLLDPE sponge material holds the potential for widespread application and commercial success. The emphasis on optimization further ensures that the large-scale production process is feasible, efficient, and economically viable. Here is an example techno-economic analysis for a commercial scale production facility manufacturing 2000 tons/year of the Ir-rLLDPE sponge material:

Assumptions:

- Plant lifetime: 10 years
- Tax rate: 25%
- Depreciation: 10 years (straight line)
- Irradiator cost: $1 million
- Thermal reactor cost: $1 million
- Operating labor: $50,000/year
- Utility costs: $100,000/year
- Raw material costs: $1.2 million/year
- General expenses: 5% of fixed capital investment/year

Total capital investment (TCI):

- Fixed capital investment (irradiator, thermal reactor): $2 million
- Working capital investment (10% of FCI): $0.2 million Total capital investment = Fixed capital + Working capital = $2 million + $0.2 million = $2.2 million

Annual production costs:

- Direct manufacturing costs (raw materials, utilities): $1.3 million/year
- Indirect manufacturing costs (labor, maintenance): $0.15 million/year
- Fixed manufacturing costs (depreciation): $0.2 million/year
- General expenses (5% of FCI): $0.1 million/year

Total annual production cost = $1.3 + $0.15 + $0.2 + $0.1 = $1.75 million.

Profit on Sales (POS):

- Sale price of sponge: $3000/ton
- Annual sales revenue (2000 tons × $3000/ton): $6 million
- Annual profit on sales = Sales revenue − Production costs = $6 million − $1.75 million = $4.25 million

Return on Investment (ROI):

- Average annual profit = $4.25 million
- ROI = (Average annual profit/TCI) × 100 = ($4.25 million/$2.2 million) × 100 = 193%

Rate of return (ROR):

- ROR = Average annual profit/TCI × 100 = $4.25 million/$2.2 million × 100 = 193%

The total capital investment (TCI) includes both the fixed capital investment (FCI) for equipment like the irradiator and thermal reactor, as well as the working capital investment (WCI) which is a fraction of the FCI to cover short-term operating costs. The annual production costs are divided into four categories—direct manufacturing costs like raw materials, indirect manufacturing costs like labor, fixed manufacturing costs like depreciation, and general expenses. Summing these gives the total cost to produce 2000 tons annually. The profit on sales is determined by taking the revenue from selling 2000 tons of sponge per year at an assumed market price of $3000/ton, and subtracting the annual production costs. Return on investment (ROI) measures the profitability of the production facility as a percentage of the capital invested. It is calculated by dividing the average annual profit by the total capital investment. The rate of return (ROR) uses the same formula as ROI, indicating the percent return expected on the capital investment based on the projected profits. The high ROI and ROR values suggest this is a highly profitable process, mainly because the sponge can be sold at a premium price due to its high oil adsorption capacity and reusability.

## Conclusion

The innovation of this study is converting plastic waste into a powerful solution for environmental threats. Our findings transform the perception of plastic from a problematic pollutant into a sustainable savior. Through an ingenious process of irradiation and thermal treatment, we have engineered wasted LLDPE into a mighty clean-up warrior—the Ir-rLLDPE sponge. Scanning the battlefield of oil spills with its porous vision, this sponge soldier swiftly soaks up to 25 times its weight in crude oil invaders. Not satisfied with oil, it also captures chemical solvents that infiltrate and contaminate waterways. And like any superhero, this polymeric sponge displays uncanny abilities. Under attack, its robust structure resists damage despite repeated use. It grows stronger with higher irradiation power, evolving its molecular forces to take on any oily adversary. Once full, a simple squeeze revives our sponge champion to fight and soak again. But the true power of this innovation dose not come by accident. Advanced spectroscopy techniques reveal the secret origins of the sponge’s strength—structural transformations at the molecular level induced by irradiation and thermal treatment. By studying adsorption kinetics and equilibrium models, we uncover the dynamics of how oily pollutants are immobilized by these material defenders. Yet this sponge hero remains an eco-friendly guardian. Originating from plastic waste, now sustained through circular rebirth, its destiny is to protect the blue planet. Our war on pollution may still rage on many fronts, but advances like the Ir-rLLDPE sponge give hope that humanity, through science and vision, can rise up with solutions. This is the beginning of a new alliance between sustainability and technology where plastic waste transforms into guardians for the Earth.

The structural modifications induced by irradiation enhance the adsorption capacity and selectivity of the Ir-(rLLDPE) sponge, making it a promising solution for removing oil and organic solvents from water systems. Further research can explore the application of this irradiated sponge in real-world scenarios and investigate its performance for a broader range of pollutants and contaminants. Structural changes in irradiated rLLDPE were investigated using FTIR spectrum analysis, revealing the presence of new peaks and indicating the involvement of castor oil in the structural transformation. Thermal DSC analysis showed a slight decrease in the melting point, enthalpy change, and area under the curve of irradiated rLLDPE, suggesting that the gamma irradiation process influences the material's thermal behavior and melting characteristics. The formation of radical groups during radiolytic reactions, such as alkyl, aryl, and acyl radicals, can modify the structure and properties of rLLDPE, resulting in changes in the melting point and enthalpy observed in DSC analysis. The irradiation process influenced the morphology of the sponge-like structure, increasing its porosity and affinity for water. The adsorption capacity of the irradiated samples varied for different organic solvents, with higher adsorption capacities observed for polar and more significant nonpolar solvents. The irradiated sponge showed promising potential as an effective adsorbent for various organic solvents, offering solutions for environmental remediation and pollution control applications. The irradiation process induced structural modifications that improved the adsorption capacity of the sponge material. The adsorption capacity varied depending on the type of oil, and higher irradiation doses generally enhanced the adsorption capacity, but an optimal dose range was observed for each oil type. The pseudo-second-order model provided a more accurate description of the adsorption kinetics, suggesting that the concentration difference and the interaction between the solvent and the sponge surface influenced the adsorption process. The Langmuir isotherm model better fits the equilibrium data, indicating the formation of monolayer adsorption of 1,1,2,2-tetrachloroethane on the Ir-(rLLDPE) sponge.

UV–visible spectroscopy analysis demonstrated the effectiveness of the Ir-(rLLDPE) sponge in removing diesel fuel from water at a trace level. The decrease in intensity of the peak at 210 nm with increasing adsorption time indicated the efficient adsorption and removal of diesel fuel by the sponge. Complete removal of diesel fuel was achieved within 20 min, highlighting the fast and high efficiency of the adsorption process. This study introduces several novel aspects of adsorbent materials for oil and organic solvent removal. The use of irradiated Ir-(rLLDPE) sponge as an effective adsorbent, the application of the pseudo-second-order model for adsorption kinetics, the analysis of Langmuir and Freundlich isotherms for equilibrium adsorption capacity, and the combination of UV–visible spectroscopy and visual observation for evaluating adsorption performance provide valuable insights into the structural, kinetic, and equilibrium aspects of the adsorption process.

### Supplementary Information

Supplementary Information.

Supplementary Video S1.

## Data Availability

All data generated or analyzed during this study available from the corresponding author on request.

## References

[1] Prathap A, Sureshan KMJAC (2017). Organogelator-cellulose composite for practical and eco-friendly marine oil-spill recovery. Angew. Chem..

[2] Zhang W (2021). Governance of global vessel-source marine oil spills: Characteristics and refreshed strategies. Ocean Coast. Manage..

[3] Zhang B (2019). World Seas: An Environmental Evaluation.

[4] Ghina F (2003). Sustainable development in small island developing states. Environ. Dev. Sustain..

[5] Soto LA, Botello AV, Licea-Durán S, Lizárraga-Partida ML, Yáñez-Arancibia A (2014). The environmental legacy of the Ixtoc-I oil spill in Campeche Sound, southwestern Gulf of Mexico. Front. Mar. Sci..

[6] Chen B, Ye X, Zhang B, Jing L, Lee K (2019). Marine oil spills—preparedness and countermeasures. World Seas Environ. Eval..

[7] Soares MO (2022). The most extensive oil spill registered in tropical oceans (Brazil): The balance sheet of a disaster. Environ. Sci. Pollut. Res..

[8] Shen M (2020). (Micro) plastic crisis: Un-ignorable contribution to global greenhouse gas emissions and climate change. J. Clean. Prod..

[9] Roufou S (2021). The (potential) impact of seasonality and climate change on the physicochemical and microbial properties of dairy waste and its management. Trends Food Sci. Technol..

[10] Singh H, Bhardwaj N, Arya SK, Khatri M (2020). Environmental impacts of oil spills and their remediation by magnetic nanomaterials. Environ. Nanotechnol. Monit. Manage..

[11] Mearns AJ (2020). Effects of pollution on marine organisms. Water Environ. Res..

[12] Akram M, Taha I, Ghobashy MM (2016). Low temperature pyrolysis of carboxymethylcellulose. Cellulose.

[13] Younis SA, Ghobashy MM, Samy M (2017). Development of aminated poly (glycidyl methacrylate) nanosorbent by green gamma radiation for phenol and malathion contaminated wastewater treatment. J. Environ. Chem. Eng..

[14] Ghobashy MM, Reheem AMA, Mazied NA (2017). Ion etching induced surface patterns of blend polymer (poly ethylene glycol–poly methyl methacrylate) irradiated with gamma rays. Int. Polym. Process..

[15] Ghobashy MM, Khafaga MR (2017). Chemical modification of nano polyacrylonitrile prepared by emulsion polymerization induced by gamma radiation and their use for removal of some metal ions. J. Polym. Environ..

[16] Bichave MS (2023). Nano-metal oxides-activated carbons for dyes removal: A review. Mater. Today Proc..

[17] Alassod A (2022). Functionalization of aminoalkylsilane-grafted cotton for antibacterial, thermal, and wettability properties. RSC Adv..

[18] Biswas S, Pal A (2021). Application of biopolymers as a new age sustainable material for surfactant adsorption: A brief review. Carbohyd. Polym. Technol. Appl..

[19] de Farias MB, Prediger P, Vieira MGA (2022). Conventional and green-synthesized nanomaterials applied for the adsorption and/or degradation of phenol: A recent overview. J. Clean. Prod..

[20] Ghobashy MM, Elhady MA (2017). Radiation crosslinked magnetized wax (PE/Fe3O4) nano composite for selective oil adsorption. Comp. Comm..

[21] Yeh SK (2021). Preparation of polypropylene/high-melt-strength PP open-cell foam for oil absorption. Polym. Eng. Sci..

[22] Alassod A, Xu G (2021). Comparative study of polypropylene nonwoven on structure and wetting characteristics. J. Text. Inst..

[23] Wang B, Liang W, Guo Z, Liu W (2015). Biomimetic super-lyophobic and super-lyophilic materials applied for oil/water separation: A new strategy beyond nature. Chem. Soc. Rev..

[24] Yue R (2022). Green biomass-derived materials for oil spill response: Recent advancements and future perspectives. Curr. Opin. Chem. Eng..

[25] Zhang T (2019). Recent progress and future prospects of oil-absorbing materials. Chin. J. Chem. Eng..

[26] Gote M, Dhila H, Muley S (2023). Advanced synthetic and bio-based sorbents for oil spill clean-up: A review of novel trends. Nat. Environ. Pollut. Technol..

[27] Asadpour R (2013). Application of sorbent materials in oil spill management: A review. Caspian J. Appl. Sci. Res..

[28] Akanbi MJ, Jayasinghe SN, Wojcik A (2021). Characterisation of electrospun PS/PU polymer blend fibre mat for oil sorption. Polymer.

[29] Alassod A, Tina H, Islam SR, Huang W, Xu GJET (2022). Using polypropylene needle punch nonwoven sorbents as the interceptor for oil in static and dynamic water experiments. Environ. Technol..

[30] Alassod A (2023). Polypropylene-chitosan sponges prepared via thermal induce phase separation used as sorbents for oil spills cleanup. Polym. Bull..

[31] Abdallah R, Juaidi A, Assad M, Salameh T, Manzano-Agugliaro F (2020). Energy recovery from waste tires using pyrolysis: Palestine as case of study. Energies.

[32] Kumar S, Singh E, Mishra R, Kumar A, Caucci S (2021). Utilization of plastic wastes for sustainable environmental management: A review. ChemSusChem.

[33] Zhang F (2021). Current technologies for plastic waste treatment: A review. J. Clean. Prod..

[34] Kijo-Kleczkowska A, Gnatowski A (2022). Recycling of plastic waste, with particular emphasis on thermal methods. Energies.

[35] Dogu O (2021). The chemistry of chemical recycling of solid plastic waste via pyrolysis and gasification: State-of-the-art, challenges, and future directions. Progress Energy Combust. Sci..

[36] Alassod A, Abedalwafa MA, Xu GJET (2021). Evaluation of polypropylene melt blown nonwoven as the interceptor for oil. Environ. Technol..

[37] Carvalho WS, Cunha IF, Pereira MS, Ataíde CH (2015). Thermal decomposition profile and product selectivity of analytical pyrolysis of sweet sorghum bagasse: Effect of addition of inorganic salts. Ind. Crops Prod..

[38] Patlolla SR (2023). A review of methane pyrolysis technologies for hydrogen production. Renew. Sustain. Energy Rev..

[39] Bhatia L, Jha H, Sarkar T, Sarangi PK (2023). Food waste utilization for reducing carbon footprints towards sustainable and cleaner environment: A review. Int. J. Environ. Res. Public Health.

[40] Sun Y, Gao P, Tian W, Guan W (2023). Green innovation for resource efficiency and sustainability: Empirical analysis and policy. Resour. Policy.

[41] Mihalakakou G (2023). Green roofs as a nature-based solution for improving urban sustainability: Progress and perspectives. Renew. Sustain. Energy Rev..

[42] Putri DN (2023). Utilizing rice straw and sugarcane bagasse as low-cost feedstocks towards sustainable production of succinic acid. Sci. Total Environ..

[43] Bradley C, Corsini L (2023). A literature review and analytical framework of the sustainability of reusable packaging. Sustain. Prod. Consumption.

[44] Rusch M, Schöggl JP, Baumgartner RJ (2023). Application of digital technologies for sustainable product management in a circular economy: A review. Bus. Strat. Environ..

[45] Deka BK, Maji TK (2011). Effect of TiO2 and nanoclay on the properties of wood polymer nanocomposite. Compos. Part A Appl. Sci. Manuf..

[46] Deka BK, Maji TK (2012). Effect of nanoclay and ZnO on the physical and chemical properties of wood polymer nanocomposite. J. Appl. Polym. Sci..

[47] Sudha G, Kalita H, Mohanty S, Nayak SK (2017). Castor oil modified by epoxidation, transesterification, and acrylation processes: Spectroscopic characteristics. Int. J. Polym. Anal. Charact..

[48] Lee J, Joo MK, Oh H, Sohn YS, Jeong B (2006). Injectable gel: Poly (ethylene glycol)–sebacic acid polyester. Polymer.

[49] Hassan MM, El-kelesh NA, Dessouki AM (2008). The effect of gamma and electron beam irradiation on the thermal and mechanical properties of injection-moulded high crystallinity poly (propylene). Polym. Compos..

[50] Gayed HM, Ghobashy MM (2023). Gamma irradiation-enhanced performance of waste LLDPE thermally transformed into advanced sponge-like material for oil decontamination. Sci. Rep..

[51] Suhaimi NAS, Muhamad F, AbdRazak NA, Zeimaran E (2022). Recycling of polyethylene terephthalate wastes: A review of technologies, routes, and applications. Polym. Eng. Sci..

[52] Schyns ZOG, Shaver MP (2021). Mechanical recycling of packaging plastics: A review. Macromol. Rapid Commun..

[53] Samanta P, Mete S, Pal S, Khan MEH, De P (2024). Synthesis, characterization, degradation and applications of vinyl polyperoxides. Polym. J..

[54] Asadi-Zaki N, Mardani H, Roghani-Mamaqani H, Wang F (2024). Stimuli-induced adjustment of spatial distribution of fluorescence resonance energy transfer dyads in smart polymers. Coord. Chem. Rev..

[55] McKellar JF (1978). Photochemistry in Dyestuffs and Polymer Technology.

[56] Tzirakis MD, Orfanopoulos M (2013). Radical reactions of fullerenes: From synthetic organic chemistry to materials science and biology. Chem. Rev..

[57] Buchalla R, Schüttler C, Bögl KW (1993). Effects of ionizing radiation on plastic food packaging materials: A review. J. Food Protect..

[58] Allayarov SR (2024). Influence of γ–radiation input dose and post-radiation high temperature shear grinding on polypropylene functional group composition. Polym. Degrad. Stabil..

[59] Vatankhah E, Abasnezhad M, Nazerian M, Barmar M, Partovinia A (2022). Thermal energy storage and mechanical performance of composites of rigid polyurethane foam and phase change material prepared by one-shot synthesis method. J. Polym. Res..

[60] Gupta A, Mohanty AK, Misra M (2022). Biocarbon from spent coffee ground and their sustainable biocomposites with recycled water bottle and bale wrap: A new life for waste plastics and waste food residues for industrial uses. Compos. Part A Appl. Sci. Manuf..

[61] Bhunia K, Sablani SS, Tang J, Rasco B (2013). Migration of chemical compounds from packaging polymers during microwave, conventional heat treatment, and storage. Comprehensive Rev. Food Sci. Food Saf..

[62] Fauser P, Vorkamp K, Strand J (2022). Residual additives in marine microplastics and their risk assessment—a critical review. Mar. Pollut. Bull..

[63] Raghu S, Archana K, Sharanappa C, Ganesh S, Devendrappa H (2015). The physical and chemical properties of gamma ray irradiated polymer electrolyte films. J. Non-Cryst. Solids.

[64] Lee J, Maddipatla MVSN, Joy A, Vogt BD (2014). Kinetics of UV irradiation induced chain scission and cross-linking of coumarin-containing polyester ultrathin films. Macromolecules.

[65] Buchanan FJ, White JR, Sim B, Downes S (2001). The influence of gamma irradiation and aging on degradation mechanisms of ultra-high molecular weight polyethylene. J. Mater. Sci. Mater. Med..

[66] Hailan S (2024). Purification of colloidal oil in water emulsions by cationic adsorbent prepared from recycled polyethylene waste. Process Saf. Environ. Protect..

[67] Zou L (2020). Superhydrophobic and superoleophilic polyethylene aerogel coated natural rubber latex foam for oil-water separation application. Polym. Test..

[68] Dutta R (2023). Removal of oils and organic solvents from wastewater through swelling of porous crosslinked poly (ethylene-co-vinyl acetate): Preparation of adsorbent and their oil removal efficiency. Mar. Pollut. Bull..

[69] Olabintan AB, Ahmed E, Al Abdulgader H, Saleh TAJE (2022). Hydrophobic and oleophilic amine-functionalised graphene/polyethylene nanocomposite for oil–water separation. Technol. Innov..

[70] Cheng Y (2018). Facile preparation of high density polyethylene superhydrophobic/superoleophilic coatings on glass, copper and polyurethane sponge for self-cleaning, corrosion resistance and efficient oil/water separation. J. Colloid Interface Sci..

[71] Alnaqbi MA, Al Blooshi AG, Greish YEJ (2020). Polyethylene and polyvinyl chloride-blended polystyrene nanofibrous sorbents and their application in the removal of various oil spills. Adv. Polym. Technol..

[72] Abdullah TA (2022). Polyethylene over magnetite-multiwalled carbon nanotubes for kerosene removal from water. Chemosphere.

[73] Chung H-K (2015). Application of Langmuir and Freundlich isotherms to predict adsorbate removal efficiency or required amount of adsorbent. J. Ind. Eng. Chem..

