# Supplementary material for: Thermal conversion of irradiated LLDPE waste into sustainable sponge-like compounds: a novel approach for efficient trace-level oil–water removal
Source: Sci Rep. 2024 Feb 28;14:4833. doi: 10.1038/s41598-024-55401-1 (PMC10899568; doi:10.1038/s41598-024-55401-1)
Supplement: Supplementary file 1 — Supplementary Information. [file 41598_2024_55401_MOESM1_ESM.pdf]

1. Experimental

1.1 Oil adsorption rate and Uptake Capacity Assessment Using Ir-(rLLDPE) Sponge-like Material.

The following equation (1) was used for uptake capacity calculation with time [1]:

$$Uptake\ capacity\ (g/g)\ \ Q = \frac{M_0 - M_S}{M_S} \quad (1)$$

Q is the oil uptake capacity (g/g), M_o is the total mass of sorbents after the oil is drained, and M_s is the mass of the dry sorbent. The results are expressed as an average of triplicate readings.

Equilibrium Sorption conditions: The contact time was extended to 60 minutes to achieve uptake equilibrium. The sorbents were weighed and their uptake capacities were calculated as described in step (iv). This experimental setup and procedure allowed for investigating the oil permeation rate and sorption performance of Ir-(rLLDPE) sponge-like material. The uptake capacities determined at different contact times provided insights into the sorbent's effectiveness in sorbing crude oil spills from water media.

1.2. Kinetic studies of oil removal using Ir-(rLLDPE) sponge-like

oil adsorption kinetics are studied using pseudo-first-order and second-order kinetic models. The weight changes of the sponges immersed in oil for various time intervals are measured. Additionally, the sponges are subjected to a squeezing process to release the absorbed oil before repeating the adsorption test. This process is repeated ten times to assess the recycling adsorption performance of the treated Ir-(rLLDPE) sponges. The first-order kinetic constant (k1) and second-order kinetic constant (k2) are determined by fitting the experimental data to the respective kinetic equations (2) and (3) [2]. Non-linear regression or other appropriate methods can be used to obtain the values of k1 and k2.

$$ln\frac{Qm}{Q_m - Q_t} = k_1 t \qquad (2)$$

$$\frac{t}{Q_t} = \frac{t}{Q_m} + \frac{1}{k_2 Q_m^2} \qquad (3)$$

Where Q_m is the maximum oil adsorption capacity (g/g) and Q_t is the oil adsorption capacity (g/g) at time t (Min).

For the recycling adsorption experiment, After the oil adsorption kinetics analysis, the oil-absorbed Ir-(rLLDPE) sponge is subjected to a squeezing process to release the absorbed oil. The squeezed Sponge is then used in the adsorption test again. This process of oil absorption, squeezing, and repeated adsorption is repeated ten times to evaluate the recycling adsorption performance of the treated Ir-

(rLLDPE) sponges. Note: The results and discussion section provide the specific experimental conditions, oil types, and detailed analysis methods for obtaining the kinetic constants.

1.3. isotherm model studies of oil removal using Ir-(rLLDPE) sponge-like

Langmuir and Freundlich isotherm models can be employed to analyze further the oil adsorption behavior of Ir-(rLLDPE) sponges [3]. These models describe the relationship between the amount of oil absorbed by the Sponge and the concentration of oil in the surrounding media. Here is an overview of the Langmuir and Freundlich isotherms in the experiment context. The Langmuir isotherm assumes a monolayer adsorption process, where the oil molecules are adsorbed onto a limited number of available sites on the sponge surface. The Langmuir isotherm is given by equation (4):

$$q_e = \frac{q_m k_L C_e}{1 + k_L C_e} \quad (4)$$

Where: q_e is the oil adsorption capacity of the Sponge (g/g), q_m is the maximum oil adsorption capacity of the Sponge (g/g), K_L is the Langmuir constant related to the affinity between the oil and the Sponge. C_e is the oil concentration in the surrounding media (g/mL), A linear expression of the Langmuir isotherm may be represented by (5):

$$\frac{C_e}{q_e} = \frac{1}{q_m k_L} + \frac{C_e}{q_m} \quad (5)$$

By plotting the experimental data of $\frac{C_e}{q_e}$ against C_e and fitting it to the Langmuir equation, the values of q_m and K_L can be determined. The q_m represents the maximum oil adsorption capacity of the Sponge, while K_L provides insights into the adsorption affinity between the Sponge and oil.

The Freundlich isotherm assumes a multilayer or heterogeneous adsorption process, where the oil molecules are adsorbed onto various sites with different affinities on the sponge surface. The Freundlich isotherm is given by equation (6):

$$q_e = K_f C_e^{1/n} \quad (6)$$

Where: K_f is the Freundlich constant related to the adsorption capacity of the Sponge, “n” is the Freundlich constant related to the intensity of adsorption. The linear form of Freundlich isotherm is expressed as:

$$log\, q_e = \left(\frac{1}{n}\right) log\, C_e + \ log\, k_f \ \ (7)$$

By plotting the experimental data of $log\, q_e$ against $log\, C_e$ and fitting it to the Freundlich equation, the values of K_f and n can be determined. K_f represents the adsorption capacity of the Sponge, while n provides insights into the adsorption intensity. The Langmuir and Freundlich isotherms can provide valuable information about the adsorption behavior and capacity of the Ir-(rLLDPE) sponges towards oil. The determination of the Langmuir and Freundlich constants can assist in understanding the adsorption mechanisms and optimizing the use of these sponges for oil sorption applications.

2. Results and discussion

2.1. Evaluate the Oil Adsorption Capacity of Ir-(rLLDPE) Sponge Obtained via Pyrolysis of Pre-Irradiated (rLLDPE) Samples

Video S1: Demonstration of Crude Oil Removal Using LLDPE Sponge-Like Materials

Video S1 serves as a visual representation of the practical application of our sponge-like materials for the rapid removal of crude oil from a water-oil mixture. The video showcases a glass column filled with LLDPE sponge-like materials, emphasizing their functional role in facilitating efficient oil-water separation.

The key highlights of Video S1 include:

1. **Experimental Setup:** The glass column is filled with the LLDPE sponge-like materials, which have been designed to exhibit selective adsorption properties for crude oil.

2. **Oil-Water Mixture Introduction:** A mixture of crude oil and water is introduced into the glass column, simulating a real-world scenario where oil and water are mixed.

3. **Rapid Oil Separation:** The video demonstrates the efficient and rapid separation of crude oil from the water phase as it is selectively adsorbed by the LLDPE sponge-like materials. This process showcases the practical effectiveness of our materials for fast oil removal.

4. **Clear Water Effluent:** As a result of the effective adsorption process, the water effluent at the bottom of the glass column is visibly clear, demonstrating the successful removal of crude oil.

Reference

1 Meng, G. *et al.* Fabrication of superhydrophobic cellulose/chitosan composite aerogel for oil/water separation. *Fibers and Polymers* **18**, 706-712 (2017).

2 Feng, J., Nguyen, S. T., Fan, Z. & Duong, H. M. Advanced fabrication and oil absorption properties of super-hydrophobic recycled cellulose aerogels. *Chemical Engineering Journal* **270**, 168-175 (2015).

3 Keshavarz, A., Zilouei, H., Abdolmaleki, A. & Asadinezhad, A. Enhancing oil removal from water by immobilizing multi-wall carbon nanotubes on the surface of polyurethane foam. *Journal of Environmental Management* **157**, 279-286, doi:https://doi.org/10.1016/j.jenvman.2015.04.030 (2015).
